# On-Admission Pressure Ulcer Prediction Using the Nursing Needs Score

**DOI:** 10.2196/medinform.3850

**Published:** 2015-02-11

**Authors:** Yoko Nakamura, A. Ammar Ghaibeh, Yoko Setoguchi, Kazue Mitani, Yoshiro Abe, Ichiro Hashimoto, Hiroki Moriguchi

**Affiliations:** ^1^Department of Medical InformaticsInstitute of Health BiosciencesThe University of Tokushima Graduate SchoolTokushimaJapan; ^2^Department of NursingTokushima University HospitalTokushimaJapan; ^3^Department of Plastic and Reconstructive SurgeryInstitute of Health BiosciencesThe University of Tokushima Graduate SchoolTokushimaJapan

**Keywords:** pressure ulcer, nursing needs score, prediction, logistic regression, imbalanced data

## Abstract

**Background:**

Pressure ulcers (PUs) are considered a serious problem in nursing care and require preventive measures. Many risk assessment methods are currently being used, but most require the collection of data not available on admission. Although nurses assess the Nursing Needs Score (NNS) on a daily basis in Japanese acute care hospitals, these data are primarily used to standardize the cost of nursing care in the public insurance system for appropriate nurse staffing, and have never been used for PU risk assessment.

**Objective:**

The objective of this study was to predict the risk of PU development using only data available on admission, including the on-admission NNS score.

**Methods:**

Logistic regression was used to generate a prediction model for the risk of developing PUs after admission. A random undersampling procedure was used to overcome the problem of imbalanced data.

**Results:**

A combination of gender, age, surgical duration, and on-admission total NNS score (NNS group B; NNS-B) was the best predictor with an average sensitivity, specificity, and area under receiver operating characteristic curve (AUC) of 69.2% (6920/100), 82.8% (8280/100), and 84.0% (8400/100), respectively. The model with the median AUC achieved 80% (4/5) sensitivity, 81.3% (669/823) specificity, and 84.3% AUC.

**Conclusions:**

We developed a model for predicting PU development using gender, age, surgical duration, and on-admission total NNS-B score. These results can be used to improve the efficiency of nurses and reduce the number of PU cases by identifying patients who require further examination.

## Introduction

### Pressure Ulcers

The National Pressure Ulcer Advisory Panel (NPUAP)/*European Pressure Ulcer Advisory Panel*, 2009 defines a pressure ulcer (PU) as a “localized injury to the skin and/or underlying tissue usually over a bony prominence, as a result of pressure, or pressure in combination with shear” [1]. PUs are also known as pressure sores, decubitus ulcers, and bedsores.

PUs result in excessive hospital lengths of stay [2] and increase treatment costs [3]. Some PUs can be prevented, thereby reducing costs considerably. Bennett et al showed that PU treatment costs would likely increase as the population ages and the incidence of pressure damage increases [4]. Particularly since Japan is an aging society (the Ministry of Health, Labour, and Welfare expects that Japan will have a globally unprecedented super-aging population by 2025) [5], this will greatly influence national medical care expenditures, thus leading to an escalating financial burden. PU prevalence is much lower in Japan than in other countries, and the Ministry of Health, Labour, and Welfare implemented PU reduction measures in all hospitals covered by the National Medical Insurance System [6]. The latest advances in data mining techniques will help predict the risk of PU development and further reduce the number of PU cases.

The Japanese Society of Pressure Ulcers considers shock, surgical duration of more than six hours, and peripheral circulatory insufficiency among the factors that contribute to a high risk of developing PUs [7]. However, no single factor explains PU risk, but rather a complex interaction of factors increases the probability of PU development [8]. Surgical intervention itself is a risk factor [9], and surgical duration is significantly positively associated with PU development [10]. Some studies have shown that PU development is negatively correlated with mobility and activity [8]; neurological, motor impairment and activity level [11]; mobility, activity, and sensory perception [12]; functional abilities such as getting out of bed, walking, and feeding [13]; and laboratory values such as albumin [14]. Yet, other studies have shown that PU development is positively correlated with length of bedfast period [15]; Nursing Needs Scores (NNS) [16]; comorbidities (eg, diabetes, chronic renal failure, congestive heart failure, and metastatic cancer) [8,14], and longer hospital stay [17]. Gender, on the other hand, is rather controversial. Some studies have reported that males are more prone to develop PUs than females [8,15], while others have reported a higher tendency among females [9]. Yet, other studies have concluded that gender is not a risk factor for PU development [17,18]. A number of studies have reported that increasing age is a risk factor [9,17,18].

Nurses usually use a risk assessment scale, such as the Waterlow or Braden scale, to identify high-risk patients, reviewed in [11]. According to a systematic review, some studies have shown that a low total Braden score is significantly positively associated with the development of PUs [8]. However, Anthony et al argued that there is no evidence that the use of risk assessment scales reduces PU incidence [19].

Tokushima University Hospital established a team of PU specialists in 2007 to detect early PU cases and prevent their advancement. An interdisciplinary team composed of plastic and reconstructive surgeons; wound, ostomy, and continence nurses; a medical informatics engineer; a physical therapist; and others have been designated for this purpose. They investigate data of PU patients and discuss countermeasures on a weekly basis. All inpatients in Tokushima University Hospital are assessed for their PU risk using a nonstandard procedure requiring the collection of additional items. High-risk inpatients are followed up according to our hospital PU risk assessment protocol with the Braden scale. A method that can easily and accurately identify high-risk patients for further inspection using only data available on admission would be highly beneficial.

**Table 1:** NNS items in general wards.

NNS-A^a^	Monitoring and treatment	Score			NNS-B^b^	Patient condition	Score		
		0	1	2			0	1	2
1	Wound treatment	No	Yes		10	Turn over	Able	Partially able	Unable
2	Blood pressure measurement	0-4 times	More than 5 times		11	Sit up	Able	Unable	
3	Urine volume measurement	No	Yes		12	Keep a sitting position	Able	Partially able	Unable
4	Respiratory care	No	Yes		13	Transfer activity	Able	Partially able	Unable
5	≥3 Intravenous lines	No	Yes		14	Oral care	Able	Unable	
6	Electrocardiogram monitor	No	Yes		15	Feed self	No assistance	Partial assistance	Full assistance
7	Syringe pump	No	Yes		16	Change clothes	No assistance	Partial assistance	Full assistance
8	Blood transfusion or blood derivative	No	Yes						
9	Specialized treatment^c^	No		Yes					

^a^ NNS-A=Nursing Needs Score-A

^b^ NNS-B=Nursing Needs Score-B

^c^ 1, antineoplastic agent; 2, narcotic injection; 3, radiation therapy; 4, immunosuppressive agent; 5, vasopressor agent; 6, antiarrhythmic agent; and 7, drainage management

### Nursing Needs Score

Many methods have been implemented for standardizing medical cost calculations to comply with the Japanese medical insurance system that includes all residents in Japan. In acute care hospitals, one method used to evaluate nursing costs is the NNS that was introduced first to intensive care units in 2003, then to high-care units in 2004, and finally to general wards in 2008 (Table 1) [20]. The NNS provides a score of 0, 1, or 2 for each nursing task. Nursing tasks are divided into two groups: (1) group A (NNS-A) includes tasks performed during patient monitoring and treatment, and (2) group B (NNS-B) includes tasks related to patient condition. The total NNS-A score ranges from 0 to 10, while the total NNS-B score ranges from 0 to 12. Table 1 shows examples of NNS-A and NNS-B tasks used in general wards. Our hospital finished implementing NNS in its hospital information system (HIS) for all divisions in 2008, and staff NNS training has been performed regularly according to Ministry of Health, Labour, and Welfare guidelines.

### Objective

Nurses are the principal specialists for risk assessment of in-hospital acquired PU. We aimed to develop a quick and simple PU prediction tool that uses only data documented by nurses on patient admission to estimate the risk of PU development. Patients identified as being at high risk can be further investigated. With this tool, there would be no need for nurses to assess factors such as a patient’s moisture, friction, or shear state using the Braden scale, or skin type, weight loss, or continence using the Waterlow scale. Rather, early predictions can be made using the data available on admission with regard to the patient’s risk of developing PUs. Those identified as being at high risk could then be identified and followed more closely.

## Methods

### Data Description

**Figure 1:**
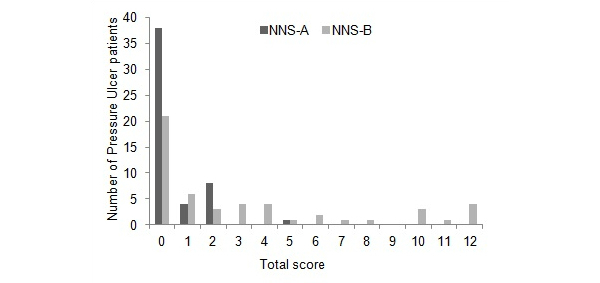
Distribution of total Nursing Needs Score group A =NNS-A, and Nursing Needs Score group B = NNS-B scores for pressure ulcers patients.

The institutional review board of Tokushima University approved the study protocol, and opt-out consent was obtained. This was a retrospective study with respect to PU prediction for all inpatients that had their NNS recorded on admission in Tokushima University Hospital (696 beds), an acute care hospital, from January 1 to December 31, 2012. This study assessed data pertaining to demographic characteristics, surgical duration, and NNS data collected from the HIS, and PU data recorded by PU specialists. The total number of PUs per patient and PU stages were categorized from “depth unknown” to Stage IV (full thickness tissue loss), as defined by NPUAP staging guidelines [1]. There were 51 patients with in-hospital PUs and 8235 control patients without in-hospital PUs. Table 2 shows the average age, average NNS-A, average NNS-B, and average surgical duration with or without PUs. Patients who did not undergo surgery were assigned 0 hours for surgery duration. Figure 1 shows the distribution of NNS-A and NNS-B scores for patients who developed PUs. NNS-B scores in particular show a broad distribution.

The data we collected suffer, as do many other medical data, from class imbalance. In an imbalanced dataset, the number of one class is much higher than the other. This occurs primarily because of a high prior probability of one class and a low prior probability of the other class. The dataset used has 8235 patients without in-hospital PU and 51 cases with in-hospital PU, resulting in a high imbalance with a positive to negative ratio of 1 to 162.

**Table 2:** Descriptive statistics of patient age.

Gender		PU-positive		PU-negative	
		n	Age mean (SD)	n	Age mean (SD)
Male		35	62.1 (14.1)	4566	62.6 (15.6)
Female		16	67.2 (14.7)	3669	59.4 (18.0)

**Table 3:** Descriptive statistics of patient surgical duration.

Surgery		PU-positive		PU-negative	
		n	Duration (hours) mean (SD)	n	Duration (hours) mean (SD)
Yes		28	6.5 (4.4)	3158	2.7 (2.2)
No		23	-	5077	-

**Table 4:** Descriptive statistics of patient NNS.

PU-positive	PU-negative
NNS-A mean (SD)	NNS-B mean (SD)
0.5 (1.0)	0.3 (0.8)
3.2 (4.0)	1.1 (2.3)

### Method

Logistic regression analysis is commonly used to determine the relationship between different qualitative and quantitative independent variables and a qualitative dependent variable. In this study, the dependent variable was whether the patient developed an in-hospital PU or not. The logistic regression model was generated using the Weka logistic regression component in RapidMiner version 5.3, Community version (RapidMiner, Inc, USA).

Standard data analysis procedures do not apply to imbalanced datasets [21], and the process of model generation and evaluation must take data imbalance into consideration. A common method for dealing with imbalanced data is to artificially balance the data during model generation (training) by either undersampling the majority class or oversampling the minority one [22]. Studies have shown that undersampling performs better than oversampling on large domains [23], and although many undersampling methods exist, the simple procedure of randomly selecting a reduced set from the majority class provides competitive results compared with other undersampling methods [24]. In this study, we used a random undersampling method during model generation to overcome the problem of class imbalance.

**Figure 2:**
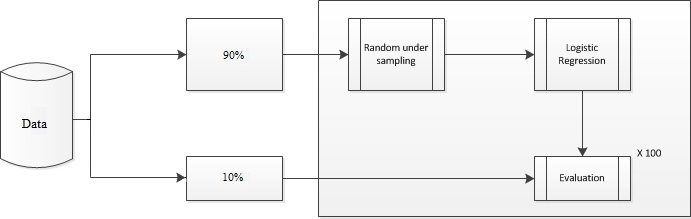
Model generation and evaluation process.

Data were randomly divided into two sets. The first set (training dataset) includes 90.00% (7412/8235 patients without in-hospital PU) and 90% (46/51 cases with in-hospital PU) of each class and was used for model generation, and the second set (test dataset) consists of the remaining 9.99% (823/8235 patients without in-hospital PU) and 9% (5/51 cases with in-hospital PU) of each class and was used to evaluate the generated model (Figure 2 shows this). This was necessary for validating the generated model on an imbalanced dataset. From the training set, we randomly sampled a smaller set of the negative class (ie, patients who did not develop in-hospital PU) and combined it with the positive class (ie, patients who developed in-hospital PU), thereby obtaining a balanced dataset. A logistic regression model was generated using the balanced dataset, and evaluated using the imbalanced dataset. This was repeated 100 times in order to cancel the effects of chance.

Using the above-described method, the following three sets of factors were examined to determine the most predictive dataset.

1. The ΣA set of factors were gender, age, surgical duration, and total NNS-A score
2. The ΣB set of factors were gender, age, surgical duration, and total NNS-B score
3. The ΣAB set of factors were gender, age, surgical duration, total NNS-A score, and total NNS-B score.

### Model Evaluation

**Figure 3:**
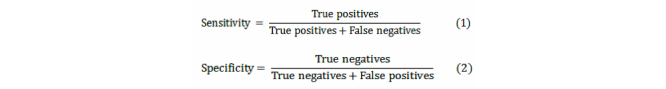
Sensitivity and specificity.

Our aim was to use the generated logistic regression model for classification, so the classification table is the most appropriate evaluation method [21]. In classification, the generated model is usually evaluated using its prediction accuracy; however, with such an imbalanced dataset, a model that simply classifies all inputs as negatives will achieve 99.38% (8235/8286) accuracy. Such a model is useless because it cannot classify the more important positive class. Therefore, other evaluation measurements are required. Common measurements used in such situations are model sensitivity (1) and specificity (2), which are defined in Figure 3.

It is important to have a model with both high sensitivity and high specificity, since a low sensitivity means the model will not efficiently predict the more important positive class, while having a low specificity means the model will have many false positives. A measurement that combines both sensitivity and specificity is the area under receiver operating characteristic curve (AUC). A high AUC indicates the model has both high sensitivity and specificity. In this study, we used AUC to identify models with high sensitivity and specificity.

## Results

### The Datasets

Table 5 shows the average accuracy, sensitivity, specificity, and AUC for each factor group over the 100 balanced datasets. The ΣA dataset gave a high average accuracy, but the average sensitivity of the generated model was very low. In contrast, the other two factor sets (ΣAB and ΣB) provided similar results with high average accuracy, sensitivity, specificity, and AUC. Given that ΣB has fewer factors, it is preferable to use ΣAB.

**Table 5:** Mean performance of different factor sets.

Factor set	Accuracy mean (SD)	Sensitivity mean (SD)	Specificity mean (SD)	AUC mean (SD)
ΣA	82.2 (2.8)	47.6 (10.5)	82.4 (2.8)	71.4 (4.5)
ΣB	82.7 (2.0)	69.2 (11.5)	82.8 (2.1)	84.0 (3.3)
ΣAB	82.1 (2.3)	70.6 (10.8)	82.2 (2.3)	84.3 (3.9)

### Models

**Figure 4:**
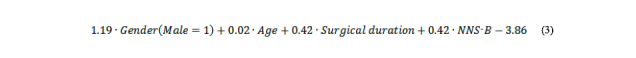
Logistic regression model of the median AUC.

The model that provided the closest results to average was the model with the median AUC (3) (Figure 4). This model achieved 80% (4/5) sensitivity, 81.3% (669/823) specificity, and 84.3% AUC. Odds ratios and 95% confidence intervals for gender, age, surgical duration, and total NNS-B score were 3.3 (1.2-10.2), 1.0 (1.0-1.1), 1.5 (1.3-1.9), and 1.5 (1.2-2.0), respectively.

It is worth mentioning that only four of the 100 models gave minimum sensitivity using the ΣB factor set, which indicates that the random undersampling procedure was effective in overcoming class imbalance and will provide a good prediction model in most cases.

## Discussion

### Predicting Pressure Ulcers at Patient Admission

We examined the possibility of predicting PU development from data typically recorded by nurses on patient admission, such as gender, age, surgical duration, and total NNS scores. Being able to achieve this would make efficient use of the existing HIS system and be of great benefit to nurses. An argument can be made that using other hospitalization data (eg, skin and support surface status, special mattresses, laboratory test results, malnutrition, etc) might improve prediction accuracy; however, our aim is to provide a system that can predict PU risk on admission without the need to collect additional data. An experienced nurse can observe changes in a patient’s status and provide an effective assessment, while a novice nurse may not be able to. With PU assessment on admission, a novice nurse can identify patients likely to develop PUs during hospitalization. Studies have shown that education [25] and use of a risk assessment scale [26] can independently influence PU prevalence.

Follow-up procedures will be needed for patients identified as being at high risk of developing PUs based on admission data. Our hospital uses the Braden scale to follow high-risk patients, which includes assessment subscales for mobility/activity risk factors [8]. To take the proper measurements, we defined PU incidence according to NPUAP and national guidelines.

Notably, gender requires further consideration. We found that, consistent with other reports [8,15], males are more prone to develop PUs than females. However, the Waterlow scoring system suggests that females are at a higher risk of PU development, and yet other studies have reported that gender does not affect the risk of developing PUs [17,18]. A potential reason for our finding that males are more prone to developing PUs is that they have less subcutaneous fat than females. In Japanese adults, a significant gender difference between males and females was found with respect to the amount of subcutaneous fat at all sites except for the side chest and lower back [27]. Given the above, the gender factor may require further investigation.

Surgical method and duration are considered important risk factors in PU development [9,10]. Our results also showed a high odds ratio for surgical duration. More than half of the inpatients that developed PUs had surgery, and most surgeries lasted more than three hours. Our results also show that the total NNS-B score is an important factor for predicting inpatient PU, while previous studies investigated the effects of individual NNS items [11-13,16].

This study showed that with highly imbalanced medical data, random undersampling could provide good results in some cases despite its simplicity. In fact, only four models generated using random undersubsampling had low sensitivity.

It is worth mentioning that no special data collection was required for this work. Collection of the data used is required at all Japanese acute care hospitals under the Japanese medical insurance system for purposes of medical fee reimbursement. Notably, this work allowed for the use of existing HIS data.

In this study, we investigated the possibility of predicting PU development in hospitalized patients from data collected by nurses on admission. Identifying the probability of developing PUs on patient admission enables nurses to take precautionary measures, and thus reduce the total number of in-hospital PU cases. The model uses the total NNS score combined with patient age, gender, and estimated surgical duration as predictive factors. Data were highly imbalanced, and the random undersampling method was used to overcome this problem. The logistic regression model achieved an average of 69.2% (6920/100) sensitivity and 82.8% (8280/100) specificity, demonstrating that random undersampling was effective for balancing the training dataset.

### Limitations

This study has a number of limitations worth noting. First, we used retrospective data with class imbalance. We are planning to investigate the results on new data. Second, the retrospective analysis was conducted in an acute care hospital setting, and thus cannot be generalized to long-term care hospitals. The current NNS does not reflect other important medical care tasks such as patient education, admission, and discharge instructions, coping with a dementia patient, fall prevention, and medication management. These are expected in a future NNS revision, and are required for further data mining of PU risk factors.

## Abbreviations

AUC: area under receiver operating characteristic curve
HIS: hospital information system
NNS: Nursing Needs Score
NPUAP: National Pressure Ulcer Advisory Panel
PU: pressure ulcers
